# Crystal structure, Hirshfeld surface analysis and DFT study of (5*E*,5′*E*,6*Z*,6′*Z*)-6,6′-[ethane-1,2-diyl­bis(aza­nylyl­idene)]bis­{5-[2-(4-fluoro­phen­yl)hydra­zono]-3,3-di­methyl­cyclo­hexa­none} 2.5-hydrate

**DOI:** 10.1107/S2056989023001895

**Published:** 2023-03-10

**Authors:** Malahat Musrat Kurbanova, Md. Serajul Haque Faizi, Emine Berrin Cinar, Asif Jamal, Mustafa Çemberci, Arzu Sadigova, Rizvan Askerov, Necmi Dege, Tahera Nabi

**Affiliations:** a Baku State University, Organic Chemistry Department, Z. Khalilov 23, Baku, AZ, 1148, Azerbaijan; b PG Department of Chemistry, Langat Singh College, B. R. A. Bihar University, Muzaffarpur, Bihar-842001, India; c Department of Physics, Faculty of Arts and Sciences, Ondokuz Mayıs University, Samsun, 55200, Türkiye; d Department of Physics, Faculty of Sciences, Ondokuz Mayıs University, Samsun, 55200, Türkiye; e Faculty of Chemistry, Karte-e-chahar, Kabul, Afghanistan; University of Durham, United Kingdom

**Keywords:** crystal structure, condensation, crystal packing, density functional theory, Hirshfeld surface analysis, hydrogen bonding

## Abstract

The title compound, C_30_H_34_F_2_N_6_O_2_·2.5H_2_O, was obtained by condensation of 2-[2-(4-fluoro­phen­yl)hydrazono]-5,5-di­methyl­cyclo­hexan-1,3-dione with ethyl­enedi­amine in ethanol and crystallized as a 1:2.5 hydrate in space group *C*2/*c*. The two independent mol­ecules, with approximate crystallographic *C*_2_ symmetries, have different conformations and packing environments, are stabilized by intra­molecular N—H⋯N hydrogen bonds and linked by O—H⋯O hydrogen bonds involving the water mol­ecules. A Hirshfeld surface analysis showed that H⋯H contacts make by far the largest (48–50%) contribution to the crystal packing. From DFT calculations, the LUMO–HOMO energy gap of the mol­ecule is 0.827 eV.

## Abstract 2

The title compound was obtained by the condensation of ethyl­enedi­amine and (5*E*,5*E*,6*Z*,6*Z*)-6,6-[ethane-1,2-diylbis(aza­nylyl­idene)]bis­{5-[2-(4-fluoro­phen­yl)hydrazono)-3,3-di­methyl­cyclo­hexa­none} in ethanol and crystallized as a 1:2.5 hydrate containing two different conformers stabilized by intra­molecular N—H⋯N and linked by O—H⋯O (involving the water mol­ecules) hydrogen bonds.

## 1. Chemical context

Diketones are versatile starting materials in the synthesis of organic and coordination compounds (Mahmudov *et al.*, 2017 ▸), used as shift reagents (Hinckley, 1969 ▸), chemical and photochemical catalysts or as biologically active derivatives to treat inflammatory diseases. β-Diketones can be isolated from natural sources such as bacteria, plants or fungi, and can also be obtained synthetically (Shokova *et al.*, 2015 ▸). More recently, 1,2-bis­(3,5-di­fluoro­phen­yl)ethane-1,2-dione has been used to synthesize various polymers for use as photovoltaics (Cai *et al.*, 2019 ▸) or gas chromatography stationary phases (Liu *et al.*, 2019 ▸). Non-covalent inter­actions, such as halogen, hydrogen, chalcogen, pnicogen, aerogen, tetrel and icosa­gen bonds, as well as π–cation, π–anion, *n*–π, π–π^*^ stacking and hydro­phobic contacts may organize or arrange the conformation and aggregation of mol­ecules, their stabilization and particular properties (Desiraju 1995 ▸; Akbari Afkhami *et al.*, 2017 ▸; Hazra *et al.*, 2018 ▸; Gurbanov *et al.*, 2018 ▸; Kvyatkovskaya *et al.*, 2017 ▸; Jlassi *et al.*, 2014 ▸). Herein we report the synthesis, crystal structure and density functional theory (DFT) calculation of the title compound (II), obtained by the condensation reaction between ethyl­enedi­amine and 2-[2-(4-fluoro­phen­yl)hydrazone]-5,5-di­methyl­cyclo­hexan-1,3-dione (I) in a 2:1 ratio, in the presence of catalytic hydro­chloric acid. A series of similar compounds was synthesized (but not characterized crystallographically) by Rema *et al.* (1997 ▸) by the condensation of 2-phenyl­hydrazones of acetyl­acetone, benzoyl­acetone and 1,3-cyclo­hexa­nedione with ethyl­enedi­amine, as well as 1,3-di­amino­propane and 1,6-di­amino­hexane. 
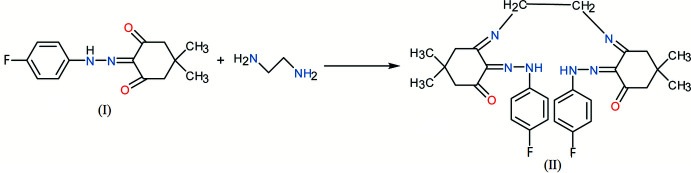


## 2. Structural commentary

The asymmetric unit contains one full mol­ecule (*A*) of (II) in a general position and one-half of another mol­ecule (*B*), which lies on a crystallographic twofold axis, as well as three ordered water mol­ecules (O1*W*, O2*W*, O3*W*) and one that is disordered over three positions (O4*W*, O5*W* and O6*W*) with occupancies of 0.5, 0.125 and 0.125, respectively. Thus the crystal composition is (II)·2.5H_2_O.

**Figure 1:**
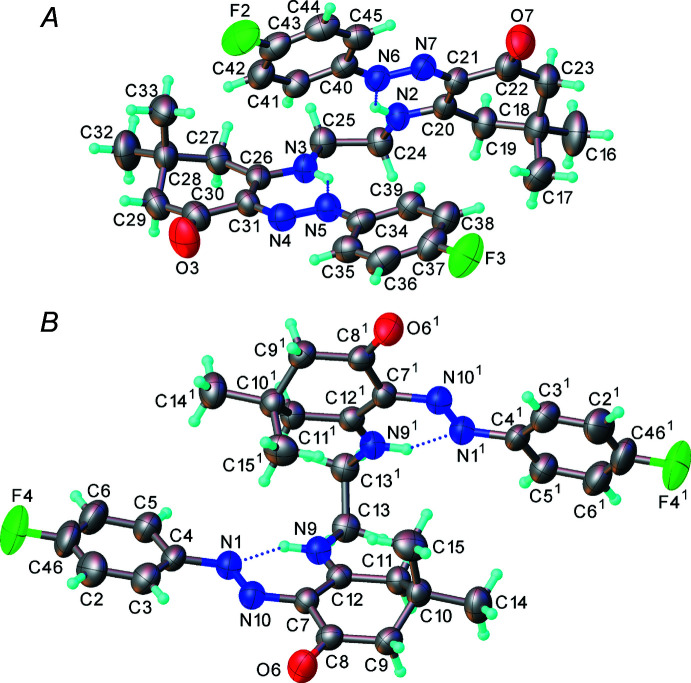
The structures of mol­ecules *A* and *B*. Displacement ellipsoids are drawn at the 40% probability level.

Mol­ecule *A* has an approximate non-crystallographic *C*_2_ symmetry. Each mol­ecule comprises two approximately planar halves, whose planarity is stabilized by intra­molecular N—H⋯N hydrogen bonds, arranged in an ‘open-book’ mode. The connecting bridges, N3—C25—C24—N2 in mol­ecule *A* and N9—C13—C13^i^—N9^i^ in *B*, have *gauche* conformations, with torsion angles of −59.1 (3) and 63.7 (3)°, respectively. However, in other respects the conformations of mol­ecules *A* and *B* are drastically different (Fig. 1 ▸). It is noteworthy that although crystal structures with *Z*′ > 1 are common, two substanti­ally different conformers rarely co-exist in the same structure (Sona & Gautham, 1992 ▸). The bond lengths in mol­ecules *A* and *B* are similar, and close to those reported earlier (Turkoglu *et al.*, 2015 ▸; Shikhaliyev *et al.*, 2019 ▸) for analogous compounds (see also Section 6).

## 3. Supra­molecular features

**Figure 2:**
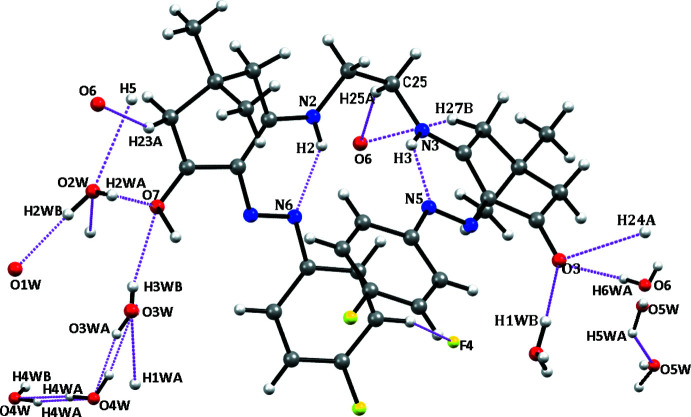
Hydrogen bonds in the crystal of (II).

In the crystal, mol­ecules of (II) are linked directly through weak C—H⋯O and C—H⋯F hydrogen bonds and indirectly through water mol­ecules of crystallization and strong O—H⋯O hydrogen bonds formed by the latter (Fig. 2 ▸, Table 1 ▸).

## 4. Hirshfeld surface analysis

**Figure 3:**
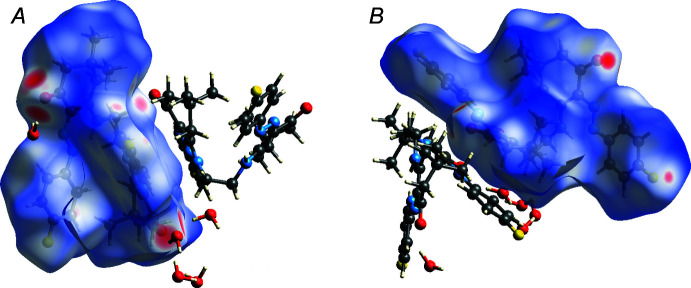
Hirshfeld surfaces of mol­ecules *A* and *B*, mapped over *d*_norm_.

In order to visualize the inter­molecular inter­actions, a Hirshfeld surface (HS) analysis (Hirshfeld, 1977 ▸) was carried out using *Crystal Explorer 17.5* (Turner *et al.*, 2017 ▸). The Hirshfeld surfaces of mol­ecules *A* and *B*, mapped over *d*_norm_, are shown in Fig. 3 ▸. The red spots, indicating short inter­molecular contacts, correspond to O—H⋯O hydrogen bonds donated by the water mol­ecules, and C—H⋯F contacts. The Hirshfeld surface of mol­ecule *B* is smaller than that of *A*, both in terms of area (527.8 *vs* 532.1 Å^2^) and the enclosed volume (684.6 *vs* 694.0 Å^3^), and has a higher asphericity factor (0.211 *vs* 0.085), as a result of the difference in conformations (see above).

**Figure 4:**
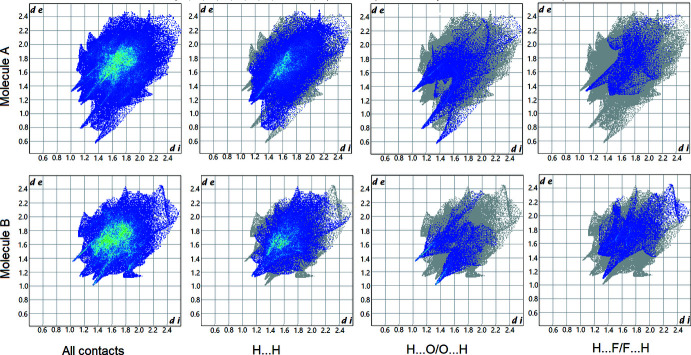
Two-dimensional fingerprint plots for (*a*) all inter­actions and delineated into (*b*) H⋯H, (*c*) H⋯O/O⋯H and (*d*) H⋯F/F⋯H contacts in mol­ecules *A* and *B*.

Two-dimensional fingerprint plots (Fig. 4 ▸) show the contributions of various contacts to the Hirshfeld surface. Thus, for both mol­ecules *A* and *B*, by far the largest contributions are by H⋯H contacts, 49.9% (*A*) and 47.9% (*B*). Given that H atoms comprise *ca* 70% of the mol­ecular surface, a similar share would be expected if the contact distribution were entirely random. The O⋯H/H⋯O contacts*, i.e.* strong and weak hydrogen bonds, contribute 14.9% (*A*) and 13.8% (*B*), and H⋯C/C⋯H contacts, *i.e.* σ–π inter­actions, 14.1% (*A*) and 11.8% (*B*). Most remarkable is the different role of the fluorine atoms. In mol­ecule *B*, F⋯H/H⋯F contacts contribute 16.8%, much more than the 6.4% in *A* or the 6% expected for a random distribution. In contrast, F⋯C/C⋯F contacts are more common in *A* (5.1%) than in *B* (1.2%). The contributions of all other contacts are negligible, except H⋯N/N⋯H (4.0% for *A*, 3.8% for *B*). Thus, the fingerprint plots reveal that mol­ecules *A* and *B* have substanti­ally different packing environments.

## 5. Frontier mol­ecular orbital analysis

The frontier mol­ecular orbitals (FMOs), *i.e*. the highest occupied MO (HOMO) and the lowest unoccupied MO (LUMO) play the most significant role in defining the mol­ecular properties (Hoffmann *et al.*, 1965 ▸; Fukui, 1982 ▸). The HOMO is associated with electron-donating and LUMO with the electron-accepting capability, their energies approximating the negative of the (first) ionization energy and the electron affinity of the mol­ecule, respectively, from where useful information regarding donor–acceptor inter­actions can be obtained (Demir *et al.*, 2016 ▸), and the degree of the electrophilicity or nucleophilicity of the mol­ecule estimated (Parr *et al.*, 1999 ▸; Chattaraj *et al.*, 2006 ▸).

**Figure 5:**
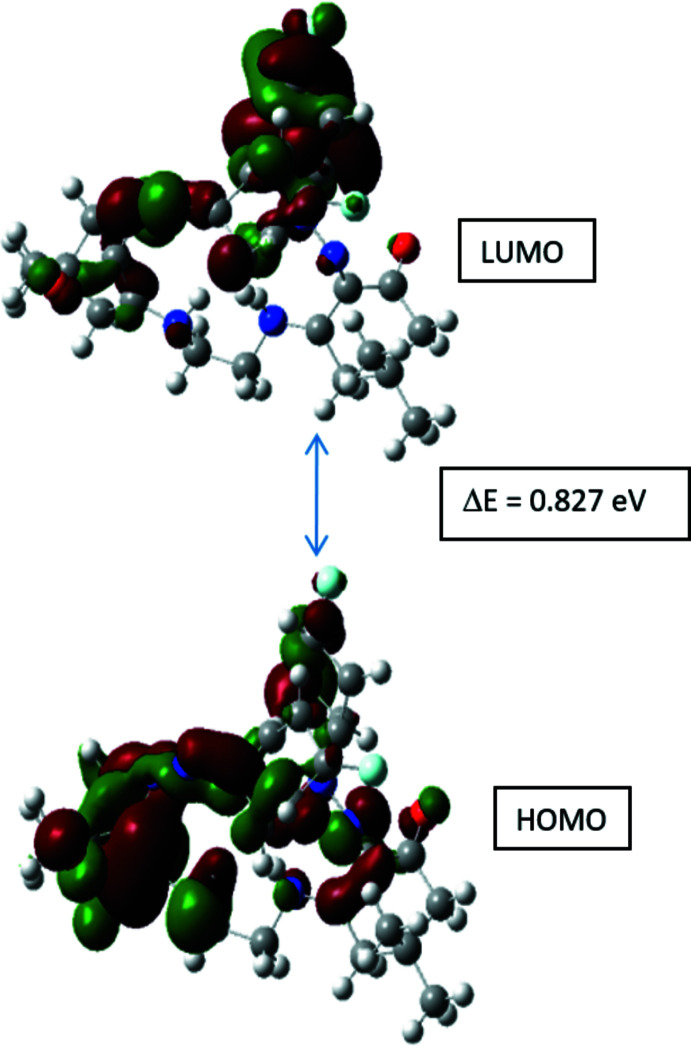
The frontier mol­ecular orbitals of (II).

The frontier orbitals of mol­ecule (II) (Fig. 5 ▸) were calculated at the DFT-B3LYP/6-311G(d,p) level of theory as implemented in *Gaussian09* (Frisch *et al.*, 2009 ▸). The X-ray-determined structure of mol­ecule *A* was taken as the starting mol­ecular geometry. This gave the energies of HOMO as −4.8164 eV and LUMO as −3.9894 eV, with a LUMO–HOMO gap of 0.827 eV, from which the chemical potential (μ = −4.40 eV), global hardness η (= 0.41 eV), softness (*S* = 1.21) and the global electrophilicity index (ω = 23.4 eV) can be derived. Thus mol­ecule (II) can be regarded as a good electrophile (Domingo *et al.*, 2002 ▸) and rather soft.

## 6. Database survey

A search of the Cambridge Database (CSD Version 5.42, update of September 2021; Groom *et al.*, 2016 ▸) did not yield any close analogue of compound (II). However, various similar compounds have been reported, *viz*. 7-(5-bromo-2-hy­droxy­phen­yl)-6-(4-bromo­phen­yl)-3,3,10,10-tetra­methyl-3,4,10,11-tetra­hydro­indolo[1,2-*a*]quinoxaline-1,8[2*H*,9*H*]-dione (ELIBIM; Fang & Yan, 2016 ▸), 6,6-dimethyl-1-(4-nitro­phen­yl)-1,5,6,7-tetra­hydro-4*H*-benzotriazol-4-one (EMOLEZ; Singh *et al.*, 2016 ▸), 3-(3-meth­oxy­phenyl­amino)-5,5-dimethyl-2-nitroso-2-cyclo­hexan-1-one (GOYFOP; Gilli *et al.*, 2000 ▸), 3-hy­droxy-6,6-dimethyl-2-(4-oxo-4*H*-chromen-3-yl)-1,5,6,7-tetra­hydro-4*H*-3,1-benzimidazol-4-one methanol solvate (ZEVJUH; Nikitina *et al.*, 2013 ▸), 1-[2,2-di­chloro-1-(4-chloro­phen­yl)ethen­yl]-2-(4-fluoro­phen­yl)diazene (XAJZIV; Nenajdenko *et al.*, 2020 ▸), 1-[(5-chloro-2-phen­oxy­phen­yl)(4-methoxy­phen­yl)carbonohydrazono­yl]-2-(4-fluoro­phen­yl)diazene (QOWNOH; Turkoglu *et al.*, 2015 ▸), (*Z*)-4-[(*E*)-(4-fluoro­phen­yl)diazen­yl]-6-[(3-hy­droxy­propyl­amino)­methyl­ene]-2-meth­oxy­cyclo­hexa-2,4-dienone (KARFAM; Albayrak *et al.*, 2012 ▸), *N*-(4-fluoro­phen­yl)-*N*′-{1-[(4-fluoro­phen­yl)di­azen­yl]-2-(methyl­imino)-2-phenyl­ethyl­idene}-2,2-di­methyl­prop­ane­hydrazide (SIDKOH; Simunek *et al.*, 2013 ▸) and 2-[2-(3-chloro-4-fluoro­phen­yl)hydrazono]-5,5-di­methyl­cyclo­hex­ane-1,3-dione (CAXPIE; Subhasri *et al.*, 2022 ▸).

## 7. Synthesis and crystallization

2 mmol of (I) were dissolved in 15–20 ml of ethanol in a three-necked flask, 1 drop of HCl was added and the solution was heated to 323 K. Then 1 mmol of ethyl­enedi­amine was added and the mixture stirred for 1 h at the same temperature. The product (II) was filtered off and purified by recrystallization from ethanol (yield 59%). The reaction and the purity of the substances were monitored by TLC (Sorbil, RF:0.72, 2-propanol).

## 8. Refinement

Crystal data, data collection and structure refinement details are summarized in Table 2 ▸. H atoms were positioned geom­etrically and refined using a riding model [O—H = 0.85, N—H = 0.86, C*sp* ^2^—H = 0.93 and C*sp* ^3^—H = 0.97 Å, *U*_iso_(H) = 1.5*U*_eq_(C) for methyl groups or 1.2*U*_eq_(O, N, C)].

## Supplementary Material

Crystal structure: contains datablock(s) I. DOI: 10.1107/S2056989023001895/zv2022sup1.cif

Structure factors: contains datablock(s) I. DOI: 10.1107/S2056989023001895/zv2022Isup3.hkl

CCDC reference: 2153643

Additional supporting information:  crystallographic information; 3D view; checkCIF report

## Figures and Tables

**Table 1:** Hydrogen-bond geometry (Å, °)

*D*—H⋯*A*	*D*—H	H⋯*A*	*D*⋯*A*	*D*—H⋯*A*
N9—H9⋯N1	0.86	1.93	2.609 (3)	135
N2—H2⋯N6	0.86	1.91	2.585 (3)	134
N3—H3⋯N5	0.86	1.90	2.582 (3)	136
C13—H13*B*⋯O2*W* ^i^	0.97	2.59	3.302 (4)	130
C25—H25*A*⋯O6	0.97	2.46	3.370 (4)	156
C24—H24*A*⋯O3^ii^	0.97	2.65	3.518 (4)	149
C24—H24*A*⋯O5*W* ^iii^	0.97	2.58	3.27 (4)	128
C27—H27*B*⋯O6	0.97	2.58	3.451 (4)	149
C42—H42⋯F4^iv^	0.93	2.52	3.218 (4)	132
C5—H5⋯O2*W*	0.93	2.53	3.441 (5)	166
C23—H23*A*⋯O6^v^	0.97	2.43	3.311 (4)	151
O2*W*—H2*WA*⋯O7	0.85	2.08	2.926 (4)	178
O2*W*—H2*WB*⋯O1*W* ^vi^	0.85	1.91	2.749 (5)	171
O1*W*—H1*WA*⋯O3*W* ^vii^	0.85	2.26	3.042 (5)	154
O1*W*—H1*WB*⋯O3	0.85	2.03	2.812 (4)	152
O3*W*—H3*WA*⋯O4*W*	0.85	2.09	2.921 (10)	164
O3*W*—H3*WA*⋯O5*W*	0.85	2.11	2.88 (4)	151
O3*W*—H3*WB*⋯O7	0.85	2.13	2.866 (4)	144
O4*W*—H4*WA*⋯O4*W* ^i^	0.85	2.20	3.04 (2)	170
O4*W*—H4*WB*⋯O3*W*	0.85	2.08	2.921 (10)	168
O5*W*—H5*WA*⋯O3*W*	0.85	2.32	2.88 (4)	124
O5*W*—H5*WB*⋯O5*W* ^i^	0.85	1.97	2.48 (7)	118
O6*W*—H6*WA*⋯O3^vi^	0.85	2.06	2.90 (2)	175

Symmetry codes: (i) 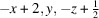 ; (II) 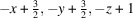 ; (iii) 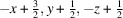 ; (iv) 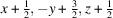 ; (v) 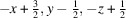 ; (vi) 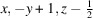 ; (vii) 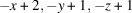 .

**Table 2:** Experimental details

Crystal data	
Chemical formula	C_30_H_34_F_2_N_6_O_2_·2.5H_2_O
*M*_r_	593.67
Crystal system, space group	Monoclinic, *C*2/*c*
Temperature (K)	296
*a*, *b*, *c* (Å)	22.7715 (19), 17.2794 (15), 25.640 (3)
β (°)	112.297 (1)
*V* (Å^3^)	9334.2 (16)
*Z*	12
Radiation type	Mo *K*α
μ (mm^−1^)	0.10
Crystal size (mm)	0.16 × 0.14 × 0.11

Data collection	
Diffractometer	Bruker APEXII CCD
No. of measured, independent and observed [*I* > 2σ(*I*)] reflections	37069, 8249, 4099
*R*_int_	0.070
(sin θ/λ)_max_ (Å^−1^)	0.596

Refinement	
*R*[*F* ^2^ > 2σ(*F* ^2^)], *wR*(*F* ^2^), *S*	0.058, 0.193, 0.99
No. of reflections	8249
No. of parameters	601
No. of restraints	25
H-atom treatment	H-atom parameters constrained
Δρ_max_, Δρ_min_ (e Å^−3^)	0.20, −0.29

Computer programs: *APEX2* and *SAINT* (Bruker, 2008 ▸), *SHELXT2013/1* (Sheldrick, 2015*a* ▸), *SHELXL2018/3* (Sheldrick, 2015*b* ▸) and *SHELXTL* (Sheldrick, 2008 ▸).

## supplementary crystallographic information

### Crystal data

**Table:**

C_30_H_34_F_2_N_6_O_2_·2.5H_2_O	*F*(000) = 3780
*M**_r_* = 593.67	*D*_x_ = 1.267 Mg m^−^^3^
Monoclinic, *C*2/*c*	Mo *K*α radiation, λ = 0.71073 Å
*a* = 22.7715 (19) Å	Cell parameters from 2620 reflections
*b* = 17.2794 (15) Å	θ = 2.5–18.8°
*c* = 25.640 (3) Å	µ = 0.10 mm^−^^1^
β = 112.297 (1)°	*T* = 296 K
*V* = 9334.2 (16) Å^3^	Prism, colourless
*Z* = 12	0.16 × 0.14 × 0.11 mm

### Data collection

**Table:**

Bruker APEXII CCD diffractometer	*R*_int_ = 0.070
φ and ω scans	θ_max_ = 25.1°, θ_min_ = 1.9°
37069 measured reflections	*h* = −27→27
8249 independent reflections	*k* = −20→20
4099 reflections with *I* > 2σ(*I*)	*l* = −30→30

### Refinement

**Table:**

Refinement on *F*^2^	25 restraints
Least-squares matrix: full	Hydrogen site location: mixed
*R*[*F*^2^ > 2σ(*F*^2^)] = 0.058	H-atom parameters constrained
*wR*(*F*^2^) = 0.193	*w* = 1/[σ^2^(*F*_o_^2^) + (0.0988*P*)^2^] where *P* = (*F*_o_^2^ + 2*F*_c_^2^)/3
*S* = 0.99	(Δ/σ)_max_ < 0.001
8249 reflections	Δρ_max_ = 0.20 e Å^−^^3^
601 parameters	Δρ_min_ = −0.29 e Å^−^^3^

### Special details

**Table:**

Geometry. All esds (except the esd in the dihedral angle between two l.s. planes) are estimated using the full covariance matrix. The cell esds are taken into account individually in the estimation of esds in distances, angles and torsion angles; correlations between esds in cell parameters are only used when they are defined by crystal symmetry. An approximate (isotropic) treatment of cell esds is used for estimating esds involving l.s. planes.

### Fractional atomic coordinates and isotropic or equivalent isotropic displacement parameters (Å^2^)

**Table:**

	*x*	*y*	*z*	*U*_iso_*/*U*_eq_	Occ. (<1)
F2	1.09554 (9)	0.57702 (14)	0.53005 (9)	0.0952 (7)	
O6	0.89907 (11)	0.85004 (13)	0.36530 (9)	0.0707 (7)	
N9	0.95271 (11)	0.63828 (14)	0.27726 (10)	0.0497 (6)	
H9	0.914706	0.638997	0.252226	0.060*	
F3	0.76710 (10)	0.32116 (12)	0.48211 (10)	0.1025 (8)	
N10	0.86237 (11)	0.75960 (14)	0.27290 (10)	0.0519 (6)	
N2	0.75280 (11)	0.67741 (14)	0.31652 (10)	0.0521 (6)	
H2	0.791352	0.677251	0.340527	0.063*	
N3	0.78027 (11)	0.76386 (14)	0.41673 (10)	0.0530 (7)	
H3	0.771048	0.715792	0.417904	0.064*	
N1	0.84455 (11)	0.71070 (15)	0.23249 (10)	0.0539 (7)	
N7	0.83957 (12)	0.55075 (15)	0.32396 (11)	0.0557 (7)	
N6	0.85905 (12)	0.60375 (15)	0.36126 (11)	0.0550 (7)	
N4	0.82821 (12)	0.67186 (16)	0.51854 (11)	0.0588 (7)	
O7	0.79621 (12)	0.44357 (15)	0.24172 (11)	0.0846 (8)	
N5	0.79668 (12)	0.63644 (15)	0.47308 (11)	0.0581 (7)	
F4	0.60711 (11)	0.74370 (16)	0.06565 (10)	0.1250 (9)	
O3	0.89386 (14)	0.75091 (17)	0.61336 (10)	0.0977 (9)	
C12	0.96907 (13)	0.69768 (16)	0.31285 (11)	0.0446 (7)	
C7	0.92362 (13)	0.75336 (17)	0.31220 (12)	0.0460 (7)	
C8	0.93933 (15)	0.80892 (17)	0.35815 (12)	0.0492 (7)	
C20	0.73494 (14)	0.61741 (17)	0.28199 (12)	0.0499 (8)	
C10	1.05603 (13)	0.78558 (17)	0.37729 (12)	0.0509 (8)	
C13	0.99160 (14)	0.57224 (16)	0.27573 (12)	0.0514 (8)	
H13A	0.968891	0.524959	0.276294	0.062*	
H13B	1.030349	0.572808	0.309210	0.062*	
C11	1.03605 (13)	0.70393 (17)	0.35377 (12)	0.0498 (7)	
H11A	1.063950	0.686857	0.335369	0.060*	
H11B	1.041915	0.669060	0.385002	0.060*	
C40	0.92104 (14)	0.59354 (18)	0.40252 (13)	0.0524 (8)	
C21	0.77828 (15)	0.55750 (17)	0.28450 (13)	0.0526 (8)	
C31	0.83760 (14)	0.75046 (19)	0.51517 (12)	0.0538 (8)	
C26	0.81638 (13)	0.79584 (17)	0.46495 (13)	0.0506 (8)	
C4	0.78281 (14)	0.72419 (19)	0.19112 (12)	0.0523 (8)	
C34	0.79058 (14)	0.55572 (19)	0.47871 (14)	0.0556 (8)	
C39	0.76263 (15)	0.5143 (2)	0.42909 (14)	0.0607 (9)	
H39	0.749549	0.540146	0.394719	0.073*	
C25	0.75416 (15)	0.80125 (18)	0.36141 (12)	0.0580 (8)	
H25A	0.788441	0.819537	0.350919	0.070*	
H25B	0.728565	0.845438	0.362886	0.070*	
C24	0.71388 (14)	0.74392 (18)	0.31816 (12)	0.0559 (8)	
H24A	0.678742	0.726986	0.327934	0.067*	
H24B	0.696646	0.768255	0.281344	0.067*	
C9	1.00665 (14)	0.81378 (18)	0.39906 (12)	0.0544 (8)	
H9A	1.010664	0.783819	0.432237	0.065*	
H9B	1.015934	0.867263	0.410773	0.065*	
C22	0.75933 (17)	0.4957 (2)	0.24298 (14)	0.0634 (9)	
C45	0.96325 (16)	0.53591 (19)	0.40083 (14)	0.0595 (9)	
H45	0.951641	0.500797	0.371137	0.071*	
C41	0.93867 (14)	0.64476 (19)	0.44682 (14)	0.0581 (8)	
H41	0.910566	0.683324	0.447703	0.070*	
C35	0.80994 (15)	0.5166 (2)	0.53017 (14)	0.0626 (9)	
H35	0.828554	0.543580	0.563835	0.075*	
C36	0.80127 (16)	0.4373 (2)	0.53080 (17)	0.0724 (10)	
H36	0.813723	0.410494	0.564756	0.087*	
C44	1.02267 (16)	0.5316 (2)	0.44389 (16)	0.0675 (10)	
H44	1.051675	0.494183	0.443113	0.081*	
C19	0.66830 (15)	0.61617 (18)	0.23968 (13)	0.0621 (9)	
H19A	0.665624	0.649403	0.208368	0.075*	
H19B	0.640637	0.637650	0.256904	0.075*	
C43	1.03795 (16)	0.5828 (2)	0.48728 (16)	0.0666 (10)	
C30	0.87277 (17)	0.7878 (2)	0.56849 (15)	0.0678 (9)	
C28	0.89082 (16)	0.90513 (19)	0.51674 (14)	0.0659 (9)	
C27	0.83332 (15)	0.87873 (17)	0.46489 (13)	0.0607 (9)	
H27A	0.796896	0.909788	0.462471	0.073*	
H27B	0.842091	0.888947	0.431352	0.073*	
C18	0.64399 (16)	0.5357 (2)	0.21674 (14)	0.0680 (10)	
C38	0.75398 (16)	0.4358 (2)	0.42995 (16)	0.0693 (10)	
H38	0.734767	0.408362	0.396538	0.083*	
C42	0.99769 (16)	0.6397 (2)	0.49014 (14)	0.0666 (9)	
H42	1.009572	0.674041	0.520310	0.080*	
C3	0.74440 (16)	0.7869 (2)	0.19078 (14)	0.0655 (9)	
H3A	0.758677	0.824248	0.218897	0.079*	
C5	0.76179 (16)	0.6708 (2)	0.14840 (14)	0.0672 (9)	
H5	0.787844	0.629339	0.148330	0.081*	
C15	1.06016 (17)	0.83952 (19)	0.33173 (14)	0.0701 (10)	
H15A	1.088826	0.818125	0.316115	0.105*	
H15B	1.075407	0.889248	0.347916	0.105*	
H15C	1.018841	0.845162	0.302490	0.105*	
C29	0.88290 (17)	0.8731 (2)	0.56898 (14)	0.0710 (10)	
H29A	0.920447	0.886039	0.601720	0.085*	
H29B	0.847052	0.898716	0.573198	0.085*	
C37	0.77395 (16)	0.3988 (2)	0.48037 (19)	0.0713 (10)	
C23	0.69345 (16)	0.4967 (2)	0.19936 (14)	0.0726 (10)	
H23A	0.680241	0.443726	0.188760	0.087*	
H23B	0.694256	0.522801	0.166131	0.087*	
C14	1.12089 (15)	0.7801 (2)	0.42564 (14)	0.0742 (10)	
H14A	1.118189	0.745758	0.454065	0.111*	
H14B	1.133661	0.830553	0.441640	0.111*	
H14C	1.151542	0.760639	0.411575	0.111*	
C46	0.66604 (18)	0.7378 (3)	0.10724 (16)	0.0812 (11)	
C6	0.70266 (19)	0.6772 (3)	0.10544 (15)	0.0791 (11)	
H6	0.688639	0.641151	0.076380	0.095*	
C33	0.95173 (17)	0.8743 (2)	0.51310 (16)	0.0831 (11)	
H33A	0.949259	0.818939	0.509510	0.125*	
H33B	0.987162	0.888138	0.546644	0.125*	
H33C	0.957199	0.896237	0.480848	0.125*	
C17	0.63175 (18)	0.4872 (2)	0.26157 (17)	0.0875 (12)	
H17A	0.670640	0.481196	0.293764	0.131*	
H17B	0.616002	0.437181	0.246323	0.131*	
H17C	0.600946	0.512764	0.272725	0.131*	
C2	0.68508 (18)	0.7934 (2)	0.14860 (16)	0.0795 (11)	
H2A	0.658518	0.834447	0.148160	0.095*	
C16	0.58150 (18)	0.5454 (2)	0.16554 (17)	0.0996 (14)	
H16A	0.550401	0.569113	0.177259	0.149*	
H16B	0.566489	0.495546	0.149430	0.149*	
H16C	0.588558	0.577506	0.137932	0.149*	
C32	0.8926 (2)	0.9931 (2)	0.51835 (18)	0.0964 (13)	
H32A	0.896750	1.012411	0.484791	0.145*	
H32B	0.928180	1.010059	0.550779	0.145*	
H32C	0.854080	1.012447	0.520502	0.145*	
O2W	0.85971 (16)	0.5119 (2)	0.17268 (14)	0.1381 (12)	
H2WA	0.840883	0.493144	0.192778	0.207*	
H2WB	0.879123	0.473704	0.165798	0.207*	
O1W	0.93443 (17)	0.6011 (2)	0.65403 (17)	0.1587 (15)	
H1WA	0.970580	0.620651	0.672785	0.238*	
H1WB	0.910130	0.639951	0.641847	0.238*	
O3W	0.92308 (17)	0.3840 (2)	0.2815 (2)	0.1847 (19)	
H3WA	0.935932	0.338346	0.279348	0.277*	
H3WB	0.882892	0.380027	0.269188	0.277*	
O4W	0.9769 (4)	0.2282 (5)	0.2984 (4)	0.180 (3)	0.5
H4WA	0.985140	0.227073	0.268724	0.270*	0.5
H4WB	0.962601	0.273503	0.298895	0.270*	0.5
O5W	0.9411 (16)	0.241 (2)	0.2313 (14)	0.175 (4)	0.125
H5WA	0.911013	0.265707	0.236069	0.263*	0.125
H5WB	0.969813	0.275397	0.236599	0.263*	0.125
O6W	0.9656 (16)	0.1682 (18)	0.2174 (11)	0.152 (11)	0.125
H6WA	0.942959	0.192682	0.187885	0.229*	0.125
H6WB	0.949669	0.123032	0.212835	0.229*	0.125

### Atomic displacement parameters (Å^2^)

**Table:**

	*U*^11^	*U*^22^	*U*^33^	*U*^12^	*U*^13^	*U*^23^
F2	0.0492 (12)	0.1286 (19)	0.0893 (15)	0.0052 (12)	0.0055 (12)	0.0275 (14)
O6	0.0634 (15)	0.0765 (16)	0.0659 (15)	0.0190 (13)	0.0174 (12)	−0.0113 (12)
N9	0.0451 (14)	0.0494 (15)	0.0520 (15)	−0.0007 (12)	0.0157 (12)	−0.0013 (13)
F3	0.0878 (16)	0.0592 (13)	0.133 (2)	−0.0079 (11)	0.0110 (14)	0.0246 (13)
N10	0.0481 (16)	0.0540 (16)	0.0504 (16)	0.0013 (13)	0.0153 (14)	0.0014 (13)
N2	0.0465 (15)	0.0554 (16)	0.0465 (15)	−0.0008 (13)	0.0088 (12)	−0.0035 (13)
N3	0.0575 (16)	0.0460 (15)	0.0480 (15)	−0.0019 (12)	0.0114 (13)	0.0015 (12)
N1	0.0471 (16)	0.0600 (17)	0.0506 (16)	−0.0003 (13)	0.0141 (14)	0.0011 (14)
N7	0.0548 (17)	0.0560 (17)	0.0556 (16)	−0.0069 (13)	0.0200 (15)	−0.0017 (14)
N6	0.0489 (16)	0.0614 (17)	0.0512 (16)	−0.0062 (13)	0.0150 (14)	−0.0006 (14)
N4	0.0586 (17)	0.0617 (19)	0.0545 (17)	0.0017 (14)	0.0194 (14)	0.0029 (14)
O7	0.0789 (18)	0.0724 (17)	0.100 (2)	−0.0005 (14)	0.0314 (16)	−0.0241 (15)
N5	0.0596 (17)	0.0575 (18)	0.0527 (17)	−0.0005 (14)	0.0162 (14)	−0.0002 (14)
F4	0.0794 (16)	0.154 (2)	0.0920 (17)	0.0178 (15)	−0.0236 (14)	0.0128 (16)
O3	0.123 (2)	0.098 (2)	0.0496 (15)	−0.0072 (17)	0.0072 (16)	0.0027 (15)
C12	0.0480 (18)	0.0432 (18)	0.0428 (17)	−0.0026 (14)	0.0174 (15)	0.0015 (14)
C7	0.0442 (18)	0.0466 (17)	0.0447 (17)	0.0040 (14)	0.0141 (15)	0.0048 (14)
C8	0.0532 (19)	0.0493 (18)	0.0453 (18)	0.0078 (16)	0.0189 (16)	0.0036 (15)
C20	0.0531 (19)	0.0517 (19)	0.0415 (17)	−0.0090 (16)	0.0143 (15)	0.0034 (15)
C10	0.0459 (18)	0.0524 (19)	0.0500 (18)	−0.0059 (15)	0.0134 (15)	−0.0055 (15)
C13	0.0559 (19)	0.0392 (16)	0.0572 (19)	−0.0019 (15)	0.0193 (16)	0.0004 (14)
C11	0.0439 (18)	0.0504 (18)	0.0518 (18)	0.0026 (15)	0.0146 (15)	0.0012 (15)
C40	0.0451 (19)	0.056 (2)	0.056 (2)	−0.0016 (16)	0.0188 (17)	0.0053 (16)
C21	0.0502 (19)	0.0482 (19)	0.056 (2)	−0.0041 (16)	0.0163 (17)	−0.0007 (16)
C31	0.0536 (19)	0.060 (2)	0.0438 (18)	0.0015 (16)	0.0141 (16)	−0.0051 (16)
C26	0.0447 (18)	0.0520 (19)	0.055 (2)	0.0034 (15)	0.0194 (16)	−0.0079 (16)
C4	0.0476 (19)	0.065 (2)	0.0389 (17)	0.0007 (17)	0.0104 (15)	0.0049 (16)
C34	0.0494 (19)	0.057 (2)	0.061 (2)	0.0031 (16)	0.0225 (17)	0.0116 (17)
C39	0.058 (2)	0.062 (2)	0.056 (2)	0.0010 (17)	0.0154 (17)	0.0074 (18)
C25	0.062 (2)	0.0522 (19)	0.0497 (19)	0.0048 (16)	0.0101 (17)	0.0060 (16)
C24	0.0528 (19)	0.057 (2)	0.0486 (18)	0.0060 (16)	0.0086 (16)	0.0040 (16)
C9	0.057 (2)	0.0558 (19)	0.0472 (18)	−0.0013 (16)	0.0162 (16)	−0.0041 (15)
C22	0.067 (2)	0.056 (2)	0.065 (2)	−0.0082 (19)	0.023 (2)	−0.0040 (18)
C45	0.061 (2)	0.061 (2)	0.061 (2)	0.0007 (18)	0.0274 (19)	0.0072 (17)
C41	0.0442 (19)	0.060 (2)	0.065 (2)	0.0024 (16)	0.0155 (18)	0.0031 (18)
C35	0.057 (2)	0.074 (2)	0.056 (2)	0.0042 (18)	0.0200 (17)	0.0082 (18)
C36	0.062 (2)	0.072 (3)	0.079 (3)	0.003 (2)	0.022 (2)	0.030 (2)
C44	0.051 (2)	0.071 (2)	0.084 (3)	0.0148 (18)	0.028 (2)	0.024 (2)
C19	0.054 (2)	0.062 (2)	0.056 (2)	−0.0027 (16)	0.0051 (17)	−0.0009 (17)
C43	0.044 (2)	0.082 (3)	0.066 (2)	−0.002 (2)	0.0114 (19)	0.021 (2)
C30	0.067 (2)	0.077 (3)	0.055 (2)	0.006 (2)	0.0184 (19)	−0.005 (2)
C28	0.065 (2)	0.061 (2)	0.066 (2)	−0.0066 (18)	0.0187 (19)	−0.0158 (18)
C27	0.064 (2)	0.053 (2)	0.061 (2)	0.0050 (16)	0.0187 (18)	−0.0070 (16)
C18	0.057 (2)	0.067 (2)	0.063 (2)	−0.0098 (18)	0.0033 (18)	−0.0026 (18)
C38	0.062 (2)	0.062 (2)	0.072 (2)	−0.0038 (18)	0.013 (2)	0.004 (2)
C42	0.052 (2)	0.075 (2)	0.065 (2)	−0.0087 (19)	0.0135 (19)	−0.0012 (19)
C3	0.067 (2)	0.068 (2)	0.055 (2)	0.0049 (19)	0.0147 (19)	0.0068 (17)
C5	0.060 (2)	0.081 (2)	0.054 (2)	0.0016 (19)	0.0142 (19)	−0.0019 (19)
C15	0.079 (2)	0.058 (2)	0.081 (2)	−0.0153 (18)	0.039 (2)	0.0003 (19)
C29	0.073 (2)	0.076 (3)	0.057 (2)	0.006 (2)	0.0171 (19)	−0.0179 (19)
C37	0.054 (2)	0.055 (2)	0.095 (3)	−0.0012 (18)	0.017 (2)	0.009 (2)
C23	0.075 (3)	0.065 (2)	0.067 (2)	−0.009 (2)	0.014 (2)	−0.0092 (18)
C14	0.057 (2)	0.077 (3)	0.077 (2)	−0.0035 (19)	0.0117 (19)	−0.016 (2)
C46	0.061 (2)	0.102 (3)	0.058 (2)	0.002 (2)	−0.004 (2)	0.012 (2)
C6	0.072 (3)	0.095 (3)	0.052 (2)	−0.005 (2)	0.004 (2)	−0.005 (2)
C33	0.063 (2)	0.104 (3)	0.077 (3)	−0.010 (2)	0.021 (2)	−0.019 (2)
C17	0.077 (3)	0.082 (3)	0.098 (3)	−0.018 (2)	0.027 (2)	0.005 (2)
C2	0.070 (3)	0.085 (3)	0.076 (3)	0.024 (2)	0.018 (2)	0.020 (2)
C16	0.075 (3)	0.091 (3)	0.091 (3)	−0.010 (2)	−0.016 (2)	−0.018 (2)
C32	0.118 (4)	0.063 (3)	0.099 (3)	−0.015 (2)	0.031 (3)	−0.025 (2)
O2W	0.137 (3)	0.153 (3)	0.134 (3)	0.008 (2)	0.062 (2)	0.002 (2)
O1W	0.130 (3)	0.116 (3)	0.188 (4)	−0.014 (2)	0.013 (3)	0.049 (2)
O3W	0.111 (3)	0.134 (3)	0.298 (6)	−0.003 (2)	0.066 (3)	0.025 (3)
O4W	0.160 (6)	0.137 (6)	0.170 (6)	−0.040 (5)	−0.019 (5)	0.015 (5)
O5W	0.157 (7)	0.138 (7)	0.166 (7)	−0.038 (6)	−0.011 (6)	0.015 (6)
O6W	0.19 (3)	0.13 (2)	0.12 (2)	0.05 (2)	0.05 (2)	0.050 (18)

### Geometric parameters (Å, º)

**Table:**

F2—C43	1.357 (4)	C36—C37	1.375 (5)
O6—C8	1.227 (3)	C36—H36	0.9300
N9—C12	1.329 (3)	C44—C43	1.360 (5)
N9—C13	1.454 (4)	C44—H44	0.9300
N9—H9	0.8600	C19—C18	1.529 (4)
F3—C37	1.353 (4)	C19—H19A	0.9700
N10—N1	1.278 (3)	C19—H19B	0.9700
N10—C7	1.380 (3)	C43—C42	1.365 (5)
N2—C20	1.323 (3)	C30—C29	1.491 (5)
N2—C24	1.461 (4)	C28—C32	1.521 (5)
N2—H2	0.8600	C28—C33	1.522 (5)
N3—C26	1.319 (3)	C28—C29	1.522 (5)
N3—C25	1.464 (3)	C28—C27	1.539 (4)
N3—H3	0.8600	C27—H27A	0.9700
N1—C4	1.423 (4)	C27—H27B	0.9700
N7—N6	1.276 (3)	C18—C23	1.517 (5)
N7—C21	1.383 (4)	C18—C17	1.531 (5)
N6—C40	1.418 (4)	C18—C16	1.536 (4)
N4—N5	1.270 (3)	C38—C37	1.357 (5)
N4—C31	1.383 (4)	C38—H38	0.9300
O7—C22	1.240 (4)	C42—H42	0.9300
N5—C34	1.414 (4)	C3—C2	1.378 (5)
F4—C46	1.365 (4)	C3—H3A	0.9300
O3—C30	1.242 (4)	C5—C6	1.383 (5)
C12—C7	1.409 (4)	C5—H5	0.9300
C12—C11	1.491 (4)	C15—H15A	0.9600
C7—C8	1.455 (4)	C15—H15B	0.9600
C8—C9	1.496 (4)	C15—H15C	0.9600
C20—C21	1.415 (4)	C29—H29A	0.9700
C20—C19	1.494 (4)	C29—H29B	0.9700
C10—C9	1.513 (4)	C23—H23A	0.9700
C10—C15	1.525 (4)	C23—H23B	0.9700
C10—C14	1.529 (4)	C14—H14A	0.9600
C10—C11	1.534 (4)	C14—H14B	0.9600
C13—C13^i^	1.507 (6)	C14—H14C	0.9600
C13—H13A	0.9700	C46—C6	1.350 (5)
C13—H13B	0.9700	C46—C2	1.373 (5)
C11—H11A	0.9700	C6—H6	0.9300
C11—H11B	0.9700	C33—H33A	0.9600
C40—C41	1.374 (4)	C33—H33B	0.9600
C40—C45	1.396 (4)	C33—H33C	0.9600
C21—C22	1.453 (4)	C17—H17A	0.9600
C31—C26	1.426 (4)	C17—H17B	0.9600
C31—C30	1.448 (4)	C17—H17C	0.9600
C26—C27	1.483 (4)	C2—H2A	0.9300
C4—C5	1.372 (4)	C16—H16A	0.9600
C4—C3	1.390 (4)	C16—H16B	0.9600
C34—C39	1.386 (4)	C16—H16C	0.9600
C34—C35	1.397 (4)	C32—H32A	0.9600
C39—C38	1.374 (4)	C32—H32B	0.9600
C39—H39	0.9300	C32—H32C	0.9600
C25—C24	1.510 (4)	O2W—H2WA	0.8500
C25—H25A	0.9700	O2W—H2WB	0.8497
C25—H25B	0.9700	O1W—H1WA	0.8499
C24—H24A	0.9700	O1W—H1WB	0.8500
C24—H24B	0.9700	O3W—H3WA	0.8500
C9—H9A	0.9700	O3W—H3WB	0.8500
C9—H9B	0.9700	O4W—H4WA	0.8503
C22—C23	1.493 (4)	O4W—H4WB	0.8499
C45—C44	1.386 (4)	O5W—H5WA	0.8501
C45—H45	0.9300	O5W—H5WB	0.8501
C41—C42	1.384 (4)	O6W—O6W^i^	1.80 (6)
C41—H41	0.9300	O6W—H6WA	0.8499
C35—C36	1.385 (5)	O6W—H6WB	0.8500
C35—H35	0.9300		
			
C12—N9—C13	127.6 (3)	C18—C19—H19B	108.7
C12—N9—H9	116.2	H19A—C19—H19B	107.6
C13—N9—H9	116.2	F2—C43—C44	118.8 (3)
N1—N10—C7	117.5 (2)	F2—C43—C42	118.2 (4)
C20—N2—C24	126.8 (3)	C44—C43—C42	123.0 (3)
C20—N2—H2	116.6	O3—C30—C31	121.8 (3)
C24—N2—H2	116.6	O3—C30—C29	119.7 (3)
C26—N3—C25	127.1 (3)	C31—C30—C29	118.5 (3)
C26—N3—H3	116.5	C32—C28—C33	109.7 (3)
C25—N3—H3	116.5	C32—C28—C29	110.5 (3)
N10—N1—C4	114.6 (3)	C33—C28—C29	110.0 (3)
N6—N7—C21	117.1 (3)	C32—C28—C27	109.0 (3)
N7—N6—C40	115.8 (3)	C33—C28—C27	109.9 (3)
N5—N4—C31	117.6 (3)	C29—C28—C27	107.8 (3)
N4—N5—C34	115.1 (3)	C26—C27—C28	114.9 (3)
N9—C12—C7	120.4 (3)	C26—C27—H27A	108.5
N9—C12—C11	119.0 (3)	C28—C27—H27A	108.5
C7—C12—C11	120.6 (3)	C26—C27—H27B	108.5
N10—C7—C12	126.6 (3)	C28—C27—H27B	108.5
N10—C7—C8	114.2 (3)	H27A—C27—H27B	107.5
C12—C7—C8	119.2 (3)	C23—C18—C19	108.4 (3)
O6—C8—C7	122.3 (3)	C23—C18—C17	110.1 (3)
O6—C8—C9	119.2 (3)	C19—C18—C17	110.6 (3)
C7—C8—C9	118.5 (3)	C23—C18—C16	110.3 (3)
N2—C20—C21	120.7 (3)	C19—C18—C16	108.2 (3)
N2—C20—C19	117.8 (3)	C17—C18—C16	109.1 (3)
C21—C20—C19	121.4 (3)	C37—C38—C39	118.9 (3)
C9—C10—C15	110.9 (3)	C37—C38—H38	120.6
C9—C10—C14	110.2 (2)	C39—C38—H38	120.6
C15—C10—C14	109.7 (3)	C43—C42—C41	118.0 (3)
C9—C10—C11	106.8 (2)	C43—C42—H42	121.0
C15—C10—C11	110.9 (2)	C41—C42—H42	121.0
C14—C10—C11	108.3 (2)	C2—C3—C4	119.7 (3)
N9—C13—C13^i^	112.5 (2)	C2—C3—H3A	120.1
N9—C13—H13A	109.1	C4—C3—H3A	120.1
C13^i^—C13—H13A	109.1	C4—C5—C6	121.4 (3)
N9—C13—H13B	109.1	C4—C5—H5	119.3
C13^i^—C13—H13B	109.1	C6—C5—H5	119.3
H13A—C13—H13B	107.8	C10—C15—H15A	109.5
C12—C11—C10	114.5 (2)	C10—C15—H15B	109.5
C12—C11—H11A	108.6	H15A—C15—H15B	109.5
C10—C11—H11A	108.6	C10—C15—H15C	109.5
C12—C11—H11B	108.6	H15A—C15—H15C	109.5
C10—C11—H11B	108.6	H15B—C15—H15C	109.5
H11A—C11—H11B	107.6	C30—C29—C28	114.9 (3)
C41—C40—C45	119.8 (3)	C30—C29—H29A	108.5
C41—C40—N6	115.6 (3)	C28—C29—H29A	108.5
C45—C40—N6	124.6 (3)	C30—C29—H29B	108.5
N7—C21—C20	126.0 (3)	C28—C29—H29B	108.5
N7—C21—C22	114.2 (3)	H29A—C29—H29B	107.5
C20—C21—C22	119.8 (3)	F3—C37—C38	119.8 (4)
N4—C31—C26	126.1 (3)	F3—C37—C36	117.7 (4)
N4—C31—C30	115.0 (3)	C38—C37—C36	122.5 (3)
C26—C31—C30	118.9 (3)	C22—C23—C18	115.6 (3)
N3—C26—C31	119.6 (3)	C22—C23—H23A	108.4
N3—C26—C27	118.3 (3)	C18—C23—H23A	108.4
C31—C26—C27	122.2 (3)	C22—C23—H23B	108.4
C5—C4—C3	119.4 (3)	C18—C23—H23B	108.4
C5—C4—N1	115.6 (3)	H23A—C23—H23B	107.4
C3—C4—N1	125.0 (3)	C10—C14—H14A	109.5
C39—C34—C35	119.2 (3)	C10—C14—H14B	109.5
C39—C34—N5	116.3 (3)	H14A—C14—H14B	109.5
C35—C34—N5	124.5 (3)	C10—C14—H14C	109.5
C38—C39—C34	120.9 (3)	H14A—C14—H14C	109.5
C38—C39—H39	119.5	H14B—C14—H14C	109.5
C34—C39—H39	119.5	C6—C46—F4	117.9 (4)
N3—C25—C24	109.4 (2)	C6—C46—C2	123.4 (3)
N3—C25—H25A	109.8	F4—C46—C2	118.7 (4)
C24—C25—H25A	109.8	C46—C6—C5	117.6 (4)
N3—C25—H25B	109.8	C46—C6—H6	121.2
C24—C25—H25B	109.8	C5—C6—H6	121.2
H25A—C25—H25B	108.2	C28—C33—H33A	109.5
N2—C24—C25	109.3 (2)	C28—C33—H33B	109.5
N2—C24—H24A	109.8	H33A—C33—H33B	109.5
C25—C24—H24A	109.8	C28—C33—H33C	109.5
N2—C24—H24B	109.8	H33A—C33—H33C	109.5
C25—C24—H24B	109.8	H33B—C33—H33C	109.5
H24A—C24—H24B	108.3	C18—C17—H17A	109.5
C8—C9—C10	115.5 (2)	C18—C17—H17B	109.5
C8—C9—H9A	108.4	H17A—C17—H17B	109.5
C10—C9—H9A	108.4	C18—C17—H17C	109.5
C8—C9—H9B	108.4	H17A—C17—H17C	109.5
C10—C9—H9B	108.4	H17B—C17—H17C	109.5
H9A—C9—H9B	107.5	C46—C2—C3	118.5 (4)
O7—C22—C21	122.5 (3)	C46—C2—H2A	120.7
O7—C22—C23	119.2 (3)	C3—C2—H2A	120.7
C21—C22—C23	118.3 (3)	C18—C16—H16A	109.5
C44—C45—C40	119.3 (3)	C18—C16—H16B	109.5
C44—C45—H45	120.4	H16A—C16—H16B	109.5
C40—C45—H45	120.4	C18—C16—H16C	109.5
C40—C41—C42	120.9 (3)	H16A—C16—H16C	109.5
C40—C41—H41	119.6	H16B—C16—H16C	109.5
C42—C41—H41	119.6	C28—C32—H32A	109.5
C36—C35—C34	119.7 (3)	C28—C32—H32B	109.5
C36—C35—H35	120.2	H32A—C32—H32B	109.5
C34—C35—H35	120.2	C28—C32—H32C	109.5
C37—C36—C35	118.9 (3)	H32A—C32—H32C	109.5
C37—C36—H36	120.6	H32B—C32—H32C	109.5
C35—C36—H36	120.6	H2WA—O2W—H2WB	104.5
C43—C44—C45	119.1 (3)	H1WA—O1W—H1WB	104.5
C43—C44—H44	120.5	H3WA—O3W—H3WB	104.5
C45—C44—H44	120.5	H4WA—O4W—H4WB	104.5
C20—C19—C18	114.3 (3)	H5WA—O5W—H5WB	104.5
C20—C19—H19A	108.7	O6W^i^—O6W—H6WA	147.7
C18—C19—H19A	108.7	O6W^i^—O6W—H6WB	107.6
C20—C19—H19B	108.7	H6WA—O6W—H6WB	104.5
			
C7—N10—N1—C4	−175.2 (2)	N7—C21—C22—C23	178.6 (3)
C21—N7—N6—C40	177.4 (2)	C20—C21—C22—C23	−1.3 (4)
C31—N4—N5—C34	177.7 (3)	C41—C40—C45—C44	−0.4 (4)
C13—N9—C12—C7	−173.7 (2)	N6—C40—C45—C44	−179.7 (3)
C13—N9—C12—C11	5.7 (4)	C45—C40—C41—C42	−0.5 (5)
N1—N10—C7—C12	−0.6 (4)	N6—C40—C41—C42	178.9 (3)
N1—N10—C7—C8	−177.4 (2)	C39—C34—C35—C36	0.4 (5)
N9—C12—C7—N10	−8.4 (4)	N5—C34—C35—C36	179.5 (3)
C11—C12—C7—N10	172.2 (3)	C34—C35—C36—C37	0.4 (5)
N9—C12—C7—C8	168.2 (2)	C40—C45—C44—C43	1.2 (5)
C11—C12—C7—C8	−11.2 (4)	N2—C20—C19—C18	−158.9 (3)
N10—C7—C8—O6	9.6 (4)	C21—C20—C19—C18	22.5 (4)
C12—C7—C8—O6	−167.4 (3)	C45—C44—C43—F2	178.3 (3)
N10—C7—C8—C9	−172.5 (2)	C45—C44—C43—C42	−1.1 (5)
C12—C7—C8—C9	10.4 (4)	N4—C31—C30—O3	1.6 (5)
C24—N2—C20—C21	178.8 (3)	C26—C31—C30—O3	−178.1 (3)
C24—N2—C20—C19	0.2 (4)	N4—C31—C30—C29	−177.7 (3)
C12—N9—C13—C13^i^	−111.6 (3)	C26—C31—C30—C29	2.7 (5)
N9—C12—C11—C10	159.0 (3)	N3—C26—C27—C28	−161.6 (3)
C7—C12—C11—C10	−21.6 (4)	C31—C26—C27—C28	18.7 (4)
C9—C10—C11—C12	51.5 (3)	C32—C28—C27—C26	−166.1 (3)
C15—C10—C11—C12	−69.4 (3)	C33—C28—C27—C26	73.8 (4)
C14—C10—C11—C12	170.2 (3)	C29—C28—C27—C26	−46.1 (4)
N7—N6—C40—C41	−171.5 (3)	C20—C19—C18—C23	−48.5 (4)
N7—N6—C40—C45	7.8 (4)	C20—C19—C18—C17	72.4 (4)
N6—N7—C21—C20	−0.6 (4)	C20—C19—C18—C16	−168.1 (3)
N6—N7—C21—C22	179.5 (3)	C34—C39—C38—C37	−0.7 (5)
N2—C20—C21—N7	5.5 (5)	F2—C43—C42—C41	−179.1 (3)
C19—C20—C21—N7	−175.9 (3)	C44—C43—C42—C41	0.3 (5)
N2—C20—C21—C22	−174.6 (3)	C40—C41—C42—C43	0.5 (5)
C19—C20—C21—C22	4.0 (4)	C5—C4—C3—C2	1.9 (5)
N5—N4—C31—C26	−1.5 (5)	N1—C4—C3—C2	−179.4 (3)
N5—N4—C31—C30	178.8 (3)	C3—C4—C5—C6	−1.1 (5)
C25—N3—C26—C31	179.9 (3)	N1—C4—C5—C6	−179.9 (3)
C25—N3—C26—C27	0.1 (4)	O3—C30—C29—C28	146.9 (3)
N4—C31—C26—N3	5.5 (5)	C31—C30—C29—C28	−33.8 (4)
C30—C31—C26—N3	−174.9 (3)	C32—C28—C29—C30	173.0 (3)
N4—C31—C26—C27	−174.8 (3)	C33—C28—C29—C30	−65.8 (4)
C30—C31—C26—C27	4.8 (4)	C27—C28—C29—C30	54.0 (4)
N10—N1—C4—C5	−177.7 (3)	C39—C38—C37—F3	−178.6 (3)
N10—N1—C4—C3	3.6 (4)	C39—C38—C37—C36	1.5 (5)
N4—N5—C34—C39	−173.0 (3)	C35—C36—C37—F3	178.8 (3)
N4—N5—C34—C35	7.8 (4)	C35—C36—C37—C38	−1.3 (5)
C35—C34—C39—C38	−0.2 (5)	O7—C22—C23—C18	153.5 (3)
N5—C34—C39—C38	−179.4 (3)	C21—C22—C23—C18	−28.2 (5)
C26—N3—C25—C24	−178.3 (3)	C19—C18—C23—C22	51.9 (4)
C20—N2—C24—C25	−176.4 (3)	C17—C18—C23—C22	−69.2 (4)
N3—C25—C24—N2	−59.1 (3)	C16—C18—C23—C22	170.2 (3)
O6—C8—C9—C10	−158.5 (3)	F4—C46—C6—C5	−178.7 (3)
C7—C8—C9—C10	23.6 (4)	C2—C46—C6—C5	1.3 (6)
C15—C10—C9—C8	68.3 (3)	C4—C5—C6—C46	−0.5 (5)
C14—C10—C9—C8	−170.0 (3)	C6—C46—C2—C3	−0.5 (6)
C11—C10—C9—C8	−52.6 (3)	F4—C46—C2—C3	179.5 (3)
N7—C21—C22—O7	−3.2 (5)	C4—C3—C2—C46	−1.2 (5)
C20—C21—C22—O7	176.8 (3)		

Symmetry code: (i) −*x*+2, *y*, −*z*+1/2.

### Hydrogen-bond geometry (Å, º)

**Table:**

*D*—H···*A*	*D*—H	H···*A*	*D*···*A*	*D*—H···*A*
N9—H9···N1	0.86	1.93	2.609 (3)	135
N2—H2···N6	0.86	1.91	2.585 (3)	134
N3—H3···N5	0.86	1.90	2.582 (3)	136
C13—H13*B*···O2*W*^i^	0.97	2.59	3.302 (4)	130
C25—H25*A*···O6	0.97	2.46	3.370 (4)	156
C24—H24*A*···O3^ii^	0.97	2.65	3.518 (4)	149
C24—H24*A*···O5*W*^iii^	0.97	2.58	3.27 (4)	128
C27—H27*B*···O6	0.97	2.58	3.451 (4)	149
C42—H42···F4^iv^	0.93	2.52	3.218 (4)	132
C5—H5···O2*W*	0.93	2.53	3.441 (5)	166
C23—H23*A*···O6^v^	0.97	2.43	3.311 (4)	151
O2*W*—H2*WA*···O7	0.85	2.08	2.926 (4)	178
O2*W*—H2*WB*···O1*W*^vi^	0.85	1.91	2.749 (5)	171
O1*W*—H1*WA*···O3*W*^vii^	0.85	2.26	3.042 (5)	154
O1*W*—H1*WB*···O3	0.85	2.03	2.812 (4)	152
O3*W*—H3*WA*···O4*W*	0.85	2.09	2.921 (10)	164
O3*W*—H3*WA*···O5*W*	0.85	2.11	2.88 (4)	151
O3*W*—H3*WB*···O7	0.85	2.13	2.866 (4)	144
O4*W*—H4*WA*···O4*W*^i^	0.85	2.20	3.04 (2)	170
O4*W*—H4*WB*···O3*W*	0.85	2.08	2.921 (10)	168
O5*W*—H5*WA*···O3*W*	0.85	2.32	2.88 (4)	124
O5*W*—H5*WB*···O5*W*^i^	0.85	1.97	2.48 (7)	118
O6*W*—H6*WA*···O3^vi^	0.85	2.06	2.90 (2)	175

Symmetry codes: (i) −*x*+2, *y*, −*z*+1/2; (ii) −*x*+3/2, −*y*+3/2, −*z*+1; (iii) −*x*+3/2, *y*+1/2, −*z*+1/2; (iv) *x*+1/2, −*y*+3/2, *z*+1/2; (v) −*x*+3/2, *y*−1/2, −*z*+1/2; (vi) *x*, −*y*+1, *z*−1/2; (vii) −*x*+2, −*y*+1, −*z*+1.
